# Trends, disparities, and pregnancy outcomes of gestational diabetes mellitus in North West London: A nine-year population-based cohort study

**DOI:** 10.1186/s12916-026-05103-4

**Published:** 2026-07-28

**Authors:** Sara Sousi, Sevasti Panagiota Glynou, Sabrina Hyman, Natasha Singh, Simon Dryden, Ara Darzi, Ana Luisa Neves

**Affiliations:** 1 Institute of Global Health Innovation, Imperial College London, London, UK; 2 School of Public Health, Imperial College London, London, UK; 3 The Royal London Hospital, Barts Health NHS Trust, London, UK; 4 Touro College of Osteopathic Medicine, New York, NY USA; 5 Chelsea and Westminster Hospital NHS Foundation Trust, London, UK; 6 Department of Metabolism, Digestion and Reproduction, Imperial College London, London, UK

**Keywords:** Gestational diabetes mellitus, Epidemiology, Real-world data, Population health, Health inequalities, Ethnic disparities, Pregnancy outcomes, North West London

## Abstract

**Background:**

Gestational diabetes mellitus (GDM) affects approximately 1 in 20 pregnancies in England. North West London (NWL) serves a diverse population of 2.4 million with substantial ethnic and socioeconomic variation. However, population-level epidemiological evidence from NWL remains limited. This study aims to characterise GDM incidence, temporal trends, risk determinants, and associated pregnancy outcomes.

**Methods:**

A retrospective population-based cohort study using routinely collected data from the Whole Systems Integrated Care database included all birth episodes from 2017 to 2025, categorised as GDM or non-GDM. Incidence per 1,000 births was calculated and temporal trends were analysed. Multivariable generalised estimating equation (GEE) logistic regression models were used to examine maternal risk determinants for GDM and pregnancy outcomes associated with GDM, while controlling for confounders and accounting for within-woman clustering across pregnancies.

**Results:**

Among 172,332 births from 134,991 women, 18,892 (10.9%) were GDM-affected. Overall incidence was 109.6 per 1,000 births (95% CI: 108.2-111.1), rising from 80.5 per 1,000 births in 2017 to 138 per 1,000 births in 2025. Incidence increased with maternal age and deprivation (119 vs. 85.4 in most vs. least deprived quintiles). Asian women had the highest risk (aOR 2.27, 95% CI: 2.15–2.40) relative to White. GDM was associated with higher rates of labour induction (51% vs. 41%), caesarean delivery (47% vs. 36%), preterm birth (7.3% vs. 5.2%), greater maternity care costs (£4,674 vs. £3,971) and longer maternal length of stay (3 vs. 2 days).

**Conclusion:**

The incidence of GDM in NWL substantially exceeds national estimates and has nearly doubled over nine years, with pronounced ethnic and socioeconomic disparities. Targeted culturally appropriate prevention strategies and equity-focused service planning are needed in this population.

**Supplementary Information:**

The online version contains supplementary material available at https://doi.org/10.1186/s12916-026-05103-4.

## Background

Gestational diabetes mellitus (GDM) is defined as diabetes first diagnosed during pregnancy that is not overt diabetes [1]. GDM affects 1 in 7 pregnancies globally, while in England, it is estimated to be 1 in 20 [2, 3]. The prevalence has been steadily increasing, which could be attributed to risk factors such as advanced maternal age and obesity [4–8]. Certain ethnic backgrounds, for example, South Asian and Middle Eastern populations, are also at higher risk [9, 10]. GDM is known to increase the risks of adverse outcomes, such as pregnancy-induced hypertension, caesarean delivery, and long-term metabolic issues like obesity and diabetes in later life for both mother and offspring [11].

In the United Kingdom, the National Institute for Health and Care Excellence (NICE) guidelines recommend screening for GDM using a risk-factor-based approach [12]. This includes women over 40, a body mass index (BMI) above 30 kg/m^2^, previous macrosomic baby, previous GDM pregnancy, family history of diabetes, history of weight-loss surgery, and if one is of South Asian, Black, African-Caribbean, or Middle Eastern origin [13]. Diagnosis is confirmed with a 75 g 2-hour oral glucose tolerance test, offered to women between 24 and 28 weeks of gestation, with GDM diagnosed if the fasting plasma glucose is 5.6 mmol/l or above, or the 2-hour plasma glucose level is 7.8 mmol/l or above [12, 14].

North West London (NWL) represents a great region for examining GDM epidemiology due to its ethnic diversity and socioeconomic heterogeneity [15]. The region is comprised of eight boroughs (Brent, Ealing, Hammersmith & Fulham, Harrow, Hillingdon, Hounslow, Kensington & Chelsea, and Westminster), serves approximately 2.4 million residents, and is one of the most ethnically diverse populations in England [16]. However, despite the recognised importance of these demographic determinants, population-level evidence describing the incidence, temporal trends, sociodemographic patterning, and obstetric outcomes of GDM within NWL remains limited.

The primary aim of this study is to characterise the incidence and temporal trends of GDM in NWL utilising the Whole Systems Integrated Care (WSIC) database. Secondary objectives are to investigate the sociodemographic inequalities associated with GDM, identify maternal risk determinants associated with GDM, and evaluate the impact of GDM on pregnancy outcomes.

## Methods

### Study design & data source

A retrospective population-based cohort study was conducted using routinely collected hospital episode statistics (HES) from the WSIC database, accessed via Discover-NOW. The WSIC dataset allows for linkage across primary, secondary care, community health, and social care records of patients registered with a general practice in NWL [16]. The database covers over 2.4 million actively registered patients, representing 95% of the NWL population. Discover-NOW provides a secure, de-identified research environment enabling analysis of linked healthcare records [17]. The study adheres to the STROBE checklist [18] (Additional file 1).

### Study population

Pregnancy episodes were identified using Office of Population Censuses and Surveys Classification of Interventions and Procedures (OPCS-4) codes (R17-R24; Additional file 2: Table S1). Eligible pregnancy episodes occurred between January 2017 and December 2025. Pregnancies were included only if women gave birth at Chelsea and Westminster NHS Foundation Trust, Hillingdon Hospitals NHS Foundation Trust, Imperial College Healthcare NHS Trust, or London North West University Healthcare NHS Trust, as these are the acute NHS Trusts for the NWL region. Pregnancies without a unique patient identifier or basic demographic information were excluded. All analyses were conducted at the pregnancy level, with each pregnancy contributing a single record regardless of singleton or multiple gestation. Basic demographic information was defined as the presence of a GP registration, which is the source of these data (date of birth, borough, IMD, and ethnicity). HES delivery data were used rather than a pregnancy register because GDM is typically diagnosed in mid-to-late pregnancy and is reliably captured in HES. Early pregnancy outcomes such as ectopic pregnancies and early miscarriages were not relevant to the study objectives and were excluded.

Within this cohort, pregnancies were classified as either GDM-affected and non-GDM pregnancies. GDM was identified using the ICD-10 diagnostic code O24.4 (“Diabetes mellitus arising in pregnancy”) recorded in the hospital spell diagnoses.

### Baseline characteristics & maternal outcomes

Maternal demographic and clinical characteristics included maternal age at delivery, smoking status, family history of diabetes, miscarriage history, BMI, Index of Multiple Deprivation (IMD) 2019 quintile, borough of residence, first language, and ethnicity. First language was classified as English based on SNOMED CT codes indicating English as the primary language. All other records were categorised as non-English or not recorded. Outcomes included type of labour (induction or spontaneous), mode of birth, polyhydramnios, postpartum haemorrhage (PPH), hypertensive disorders during pregnancy (gestational hypertension [GHTN], pre-eclampsia, eclampsia), preterm birth (< 37 weeks), length of hospital stay, and cost of hospital spell. Using prescription data, GDM treatment modality data were extracted and categorised as diet-controlled (in the absence of pharmacological prescriptions), metformin, insulin, and combined insulin/metformin therapy. The SNOMED CT, ICD-10, and OPCS-4 codes used to define characteristics and pregnancy outcomes are provided in Additional file 2: Tables S1-7.

### Statistical analysis

Maternal characteristics and pregnancy outcomes were summarised using frequencies and percentages for categorical variables and means with standard deviations (SD) or median with interquartile ranges (IQR) for normally and non-normally distributed variables, respectively. Between-group comparisons were performed using Pearson’s chi-squared test for categorical variables and the Wilcoxon rank-sum test for continuous variables, as appropriate. Incidence rates of GDM were calculated per 1,000 births with corresponding 95% confidence intervals (CIs). Population attributable fractions (PAFs) were calculated for ethnicity, age, and IMD to estimate the proportion of GDM cases in the population attributable to each factor, accounting for both its prevalence and associated risk. Temporal trends in GDM incidence were examined using time-series analyses.

Crude and adjusted generalised estimating equations (GEE) with a binomial family and exchangeable correlation structure were used to model independent risk factors for GDM and obstetric outcomes, accounting for within-woman clustering arising from multiple pregnancies contributed by the same woman. GEE was selected over standard logistic regression given that 23.9% of women contributed more than one pregnancy during the study period. Population-averaged odds ratios (ORs) are reported alongside 95% CIs and p-values. Variables with high missing rates were included in crude models only and excluded from adjusted models, with complete-case analyses used for all remaining covariates. Effect modification was assessed by including multiplicative interaction terms between GDM status and maternal age at delivery, ethnicity, and IMD quintile within the adjusted models. The statistical significance of interactions was evaluated using Wald tests, with p-value corrected for multiple comparisons using the false discovery rate. Sensitivity analyses were performed by GDM status, comparing first-time GDM with non-GDM pregnancies, and first-time GDM with recurrent GDM. GEE models were stratified by BMI (≥ 30 vs. < 30 kg/m²) and deprivation (high, IMD quintiles 1–2, vs. low, IMD quintiles 3–5).

Analyses were performed using R (version 4.4.1) [19].

### Ethical approval

Approvals and permissions to access the WSIC database were granted by the Northwest London Data Access Committee on 19 June 2025 (ID-467).

## Results

Within the WSIC database, 234,394 unique birth spells were identified. Sequential application of exclusion criteria yielded a final cohort of 172,332 births. Exclusions included: non-NWL hospital deliveries (*n* = 34,740), missing patient identifiers (*n* = 3,304), lack of GP registration (*n* = 8,287), conflicting birth method coding (*n* = 1,330), and residence outside NWL (*n* = 14,401). The final cohort represented 73.5% of the initially identified births (Additional file 2: Figure S1).

### Study cohort characteristics

A total of 172,332 births were identified across 134,991 women, of which 18,892 (10.9%) were affected by GDM. Women with GDM were older (33.7 ± 5.1 vs. 31.7 ± 5.4; *p* < 0.001), more likely to be Asian (49% vs. 32%, *p* < 0.001), and less likely to be White (23% vs. 42%), Black (11%) or mixed (3.1%). Overall, women with GDM had higher BMIs, with 15.4% having BMI ≥ 30 kg/m² compared with 7.6% in the non-GDM group (*p* < 0.001). A family history of diabetes was significantly higher in the GDM group (51% vs. 28%, *p* < 0.001), as was prior miscarriage (17% vs. 13%, *p* < 0.001). A breakdown of the characteristics is provided in Table 1. Of those with GDM, the majority were managed with diet alone (71.0%), followed by metformin (15.0%), combined insulin and metformin (8.0%), and insulin alone (5.9%). Significant differences were observed across treatment groups in age at delivery, ethnicity, first language, BMI, IMD, borough, smoking status, family history of diabetes, and miscarriage history (all *p* < 0.001) (Additional file 2: Table S8).

**Table 1:** Baseline Sociodemographic and Clinical Characteristics of Births

	Non-GDM	GDM	*p*-value²
	*N* = 153,440¹	*N* = 18,892¹	
**Sociodemographic Characteristics**			
**Age at Delivery (years)**	31.7 (5.4)	33.7 (5.1)	**< 0.001**
**Ethnicity**			**< 0.001**
Asian	49,358 (32.2%)	9,246 (48.9%)	
Black	14,893 (9.7%)	2,001 (10.6%)	
Mixed	5,842 (3.8%)	594 (3.1%)	
Not Recorded	42 (< 0.1%)	< 5 (< 0.1%)	
Other	19,374 (12.6%)	2,660 (14.1%)	
White	63,931 (41.7%)	4,387 (23.2%)	
**First Language**			**< 0.001**
English	59,703 (38.9%)	6,046 (32.0%)	
Not English	63,622 (41.5%)	9,163 (48.5%)	
Not Recorded	30,115 (19.6%)	3,683 (19.5%)	
**IMD 2019 Quintile**			**< 0.001**
1 (Most deprived)	20,631 (13.4%)	2,784 (14.7%)	
2	53,230 (34.7%)	7,096 (37.6%)	
3	43,922 (28.6%)	5,484 (29.0%)	
4	26,141 (17.0%)	2,639 (14.0%)	
5 (Least deprived)	9,516 (6.2%)	889 (4.7%)	
**Borough**			**< 0.001**
Brent	24,723 (16.1%)	3,442 (18.2%)	
Ealing	28,991 (18.9%)	3,987 (21.1%)	
Hammersmith and Fulham	14,032 (9.1%)	1,117 (5.9%)	
Harrow	17,649 (11.5%)	2,466 (13.1%)	
Hillingdon	27,575 (18.0%)	3,168 (16.8%)	
Hounslow	22,859 (14.9%)	3,115 (16.5%)	
Kensington and Chelsea	8,241 (5.4%)	606 (3.2%)	
Westminster	9,370 (6.1%)	991 (5.2%)	
**Clinical Characteristics**			
**BMI** **(kg/m**^**2**^**)**			**< 0.001**
< 18.5	16,765 (10.9%)	1,383 (7.3%)	
18.5–25	74,015 (48.2%)	7,994 (42.3%)	
25–30	6,981 (4.5%)	1,187 (6.3%)	
30+	11,711 (7.6%)	2,908 (15.4%)	
Missing	43,968 (28.7%)	5,420 (28.7%)	
**Smoking Status**			**< 0.001**
Ex-smoker	12,155 (7.9%)	1,175 (6.2%)	
Non-smoker	113,355 (73.9%)	15,475 (81.9%)	
Smoker	14,717 (9.6%)	1,265 (6.7%)	
Unknown	13,213 (8.6%)	977 (5.2%)	
**Family History of Diabetes**	42,183 (27.5%)	9,696 (51.3%)	**< 0.001**
**History of Miscarriage**	19,797 (12.9%)	3,118 (16.5%)	**< 0.001**

Abbreviations: GDM = Gestational Diabetes Mellitus; BMI = Body Mass Index; IMD = Index of Multiple Deprivation

^*1*^ *n(%) or Mean (SD);* ^*2*^ *Wilcoxon Rank Sum Test; Pearson Chi-Square Test*

### Incidence and temporal trends

**Fig. 1:**
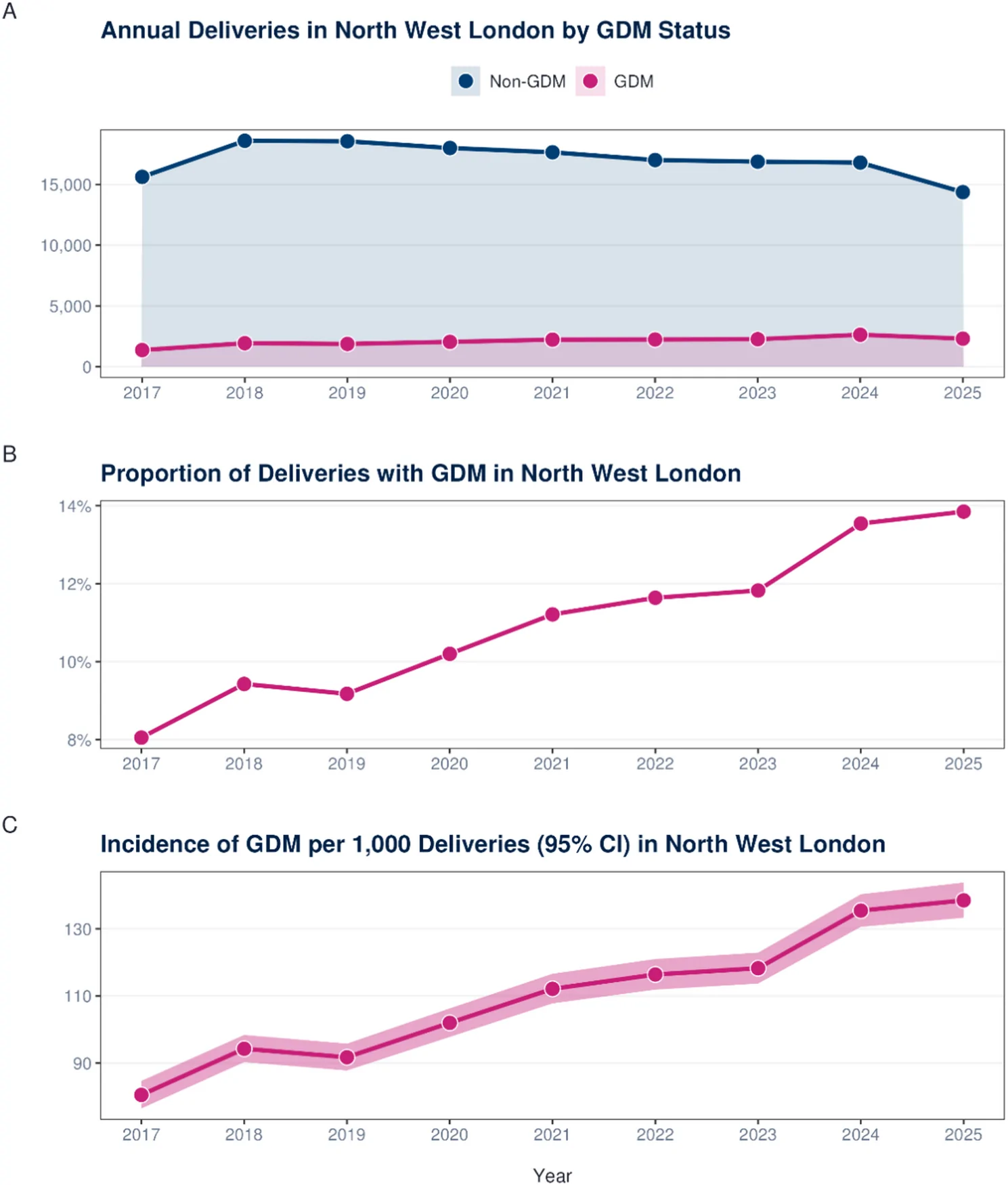
Trends in gestational diabetes mellitus (GDM) in North West London **(A)** Annual number of pregnancies by GDM status; **(B)** Proportion of all pregnancies affected by GDM; **(C)** Incidence of GDM per 1,000 pregnancies with 95% confidence intervals (CI)

The overall incidence rate of GDM-affected pregnancies was 109.6 per 1,000 births (95% CI 108.2-111.1) over the 9-year period. Since 2017, there has been a slight decline in the number of pregnancies per year across NWL with an increase in GDM diagnosis (1,368 in 2017 to 2,309 in 2025, Fig. 1A). The proportion of GDM pregnancies increased progressively from approximately 8% in 2017 to around 14% by 2025, representing almost a two-fold increase (Fig. 1B). Similarly, the incidence rate increased from roughly 81 cases per 1,000 births in 2017 to 138 per 1,000 births by 2025 (Fig. 1C).

### Incidence by age

Incidence rate per 1,000 births steeply increased with advancing maternal age (*p* < 0.001) (Table 2). Among women aged under 20 years, the incidence rate was 28.5 per 1,000 births (95% CI 21.7–36.8). Rates rose progressively across successive age categories, reaching 192 per 1,000 births (95% CI 185–200) for those aged 40–44 years and 222 per 1,000 births (95% CI 191–250) for those aged 45 years and over. Compared to women < 20, this represents an almost eight-fold higher rate.

### Incidence by geographical variation

Substantial geographical variation was observed across boroughs (*p* < 0.001) (Table 2). The highest GDM incidence rates (120–123 per 1,000 births) were recorded in Harrow, Brent, Ealing, and Hounslow. In contrast, substantially lower GDM rates were observed in Hammersmith & Fulham (73.7 per 1,000; 95% CI 69.6–78.0) and Kensington and Chelsea (68.5 per 1,000; 95% CI 63.3–74.0). Overall, GDM incidence varied by almost two-fold between the highest- and lowest-burden boroughs.

**Table 2:** Unadjusted GDM Incidence Stratified by Maternal Age, Borough, and Deprivation Index

		Total Births	GDM Affected	Incidence Rate per 1,000 Births (95% CI)
**Maternal Age at Delivery (years)**	< 20	1,999	57	28.5 (21.7–36.8)
	20–24	16,922	847	50.1 (46.8–53.4)
	25–29	41,272	3,409	82.6 (80.0–85.3)
	30–34	60,296	6,619	110.0 (107.0–112.0)
	35–39	40,971	5,847	143.0 (139.0–146.0)
	40–44	10,084	1,940	192.0 (185.0–200.0)
	45+	788	173	222.0 (191.0–250.0)
**Borough**	Brent	28,165	3,442	122.0 (118.0–126.0)
	Ealing	32,978	3,987	121.0 (117.0–124.0)
	Hammersmith & Fulham	15,149	1,117	73.7 (69.6–78.0)
	Harrow	20,115	2,466	123.0 (118.0–127.0)
	Hillingdon	30,743	3,168	103.0 (99.7–106.0)
	Hounslow	25,974	3,115	120.0 (116.0–124.0)
	Kensington and Chelsea	8,847	606	68.5 (63.3–74.0)
	Westminster	10,361	991	95.9 (90.1–102.0)
**IMD 2019 Quintile**	1 (Most deprived)	23,415	2,784	119.0 (114.0–123.0)
	2	60,326	7,096	118.0 (115.0–120.0)
	3	49,406	5,484	111.0 (108.0–114.0)
	4	28,780	2,639	91.7 (88.4–95.1)
	5 (Least deprived)	10,405	889	85.4 (80.1–91.0)

Abbreviations: GDM = Gestational Diabetes Mellitus; IMD = Index of Multiple Deprivation; CI = Confidence Interval

### Incidence by deprivation index

A positive association was observed between socioeconomic deprivation and GDM incidence across IMD quintiles (Table 2), with greater deprivation significantly associated with greater GDM burden (*p* < 0.001). Women residing in the most deprived quintile experienced the highest incidence, at 119 per 1,000 births (95% CI 114–123), with similarly elevated rates in the second quintile and third quintiles. The lowest incidence was observed in the least deprived quintile, at 85.4 per 1,000 births (95% CI 80.1–91.0), representing a relative reduction of approximately 28% compared with the most deprived group.

PAFs varied substantially across groups (Additional file 2: Table S9). White ethnicity accounted for the largest share (41.4%, 95% CI 40.5–42.3), followed by the Asian group (22.6%, 95% CI 22.2–23.1), with the remaining ethnic categories contributing negligible or negative fractions. Attributable burden also rose with advancing maternal age, from negative values in those aged under 35 to 9.4% (95% CI 9.2–9.7) at 35 to 39 years, while the most deprived IMD quintiles accounted for a greater share than the less deprived quintiles (7.2% vs. 2.6%).

### Determinants of maternal risk factors for GDM

GEE logistic regression revealed maternal age, ethnicity, language, BMI, IMD, smoking status, family history of diabetes, and prior miscarriage as independent maternal risk factors predictive of GDM (Table 3). Increasing maternal age showed a strong dose-response relationship, with each additional year adding an 8% increase in the odds (OR = 1.08, 95% CI 1.07–1.09). Asian women were at the highest risk (OR = 2.73, 95% CI 2.63–2.84) compared to White women. BMI ≥ 30 had a three-fold increase in odds (OR = 3.01, 95% CI 2.81–3.22) compared to BMI < 18.5. Notably, higher deprivation (IMD quintile 1) was associated with increased GDM risk. Current smoking reduced odds of GDM, however, smokers differed systematically from non-smokers, particularly in maternal age and ethnic distribution (Additional file 2: Table S10). Family history of diabetes and prior miscarriage independently increased risk (OR = 2.78, 95% CI 2.70–2.87 and OR = 1.33, 95% CI 1.28–1.39, respectively). After adjusting for confounders, age (aOR = 1.07, 95% CI 1.07–1.08), non-White ethnicities, family history of diabetes, a history of miscarriage, and higher BMIs were associated with increased risk of GDM, while less deprived IMD quintiles and smokers were associated with a decrease in risk.

**Table 3:** GEE Logistic Regression Analysis of Risk Factors Associated with GDM

	*N*	OR (95% CI)	*p*-value	aOR (95% CI)*	*p*-value
**Age at Delivery (years)**	172,332	1.08 (1.07, 1.09)	**< 0.001**	1.07 (1.07, 1.08)	**< 0.001**
**Ethnicity**	172,332				
White		[Ref]		[Ref]	
Asian		2.73 (2.63, 2.84)	**< 0.001**	2.27 (2.15, 2.40)	**< 0.001**
Black		1.96 (1.85, 2.07)	**< 0.001**	1.45 (1.34, 1.57)	**< 0.001**
Mixed		1.48 (1.35, 1.62)	**< 0.001**	1.27 (1.12, 1.43)	**< 0.001**
Other		2.00 (1.90, 2.10)	**< 0.001**	1.79 (1.66, 1.92)	**< 0.001**
**First Language**	138,534				
English		[Ref]		[Ref]	
Not English		1.42 (1.37, 1.47)	**< 0.001**	1.29 (1.24, 1.35)	**< 0.001**
**IMD 2019 Quintile**	172,332				
1 (Most deprived)		[Ref]		[Ref]	
2		0.99 (0.94, 1.04)	0.609	0.96 (0.90, 1.02)	0.195
3		0.93 (0.88, 0.97)	**0.002**	0.92 (0.85, 0.98)	**0.011**
4		0.75 (0.71, 0.79)	**< 0.001**	0.82 (0.76, 0.88)	**< 0.001**
5 (Least deprived)		0.69 (0.64, 0.75)	**< 0.001**	0.76 (0.68, 0.84)	**< 0.001**
**BMI (kg/m** ^**2**^ **)**	122,944				
<18.5		[Ref]		[Ref]	
18.5–25		1.31 (1.23, 1.39)	**< 0.001**	1.18 (1.10, 1.26)	**< 0.001**
25–30		2.06 (1.90, 2.24)	**< 0.001**	1.69 (1.53, 1.85)	**< 0.001**
30+		3.01 (2.81, 3.22)	**< 0.001**	2.41 (2.22, 2.61)	**< 0.001**
**Smoking Status**	158,142				
Non-smoker		[Ref]		[Ref]	
Ex-smoker		0.71 (0.67, 0.75)	**< 0.001**	0.91 (0.84, 0.99)	**0.030**
Smoker		0.63 (0.59, 0.67)	**< 0.001**	0.92 (0.85, 0.99)	**0.036**
**Family History of Diabetes**	172,332				
No		[Ref]		[Ref]	
Yes		2.78 (2.70, 2.87)	**< 0.001**	2.22 (2.13, 2.31)	**< 0.001**
**History of Miscarriage**	172,332				
No		[Ref]		[Ref]	
Yes		1.33 (1.28, 1.39)	**< 0.001**	1.09 (1.04, 1.16)	**0.001**

* Adjusted for age at delivery, ethnicity, first language, IMD 2019 quintile, BMI, smoking status, family history of diabetes, and history of miscarriage

Abbreviations: BMI = Body Mass Index; IMD = Index of Multiple Deprivation; OR = Odds Ratio; CI = Confidence Interval

### Pregnancy outcomes

GDM pregnancies had markedly different delivery patterns compared to non-GDM (Table 4). Women with GDM were more likely to undergo induction of labour (50.9% vs. 41.4%; aOR = 1.52, 95% CI 1.47–1.57) and to deliver by caesarean section, both elective (20.2% vs. 14.0%; aOR = 1.38, 95% CI 1.32–1.44) and emergency (26.6% vs. 22.2%; aOR = 1.16, 95% CI 1.12–1.20). While spontaneous vaginal delivery was less common (40.9% vs. 49.8%; aOR = 0.80, 95% CI 0.77–0.82), as was assisted vaginal birth (aOR = 0.90, 95% CI 0.86–0.94).

**Table 4:** Pregnancy Outcomes by GDM Status

	Non-GDM	GDM	OR (95% CI)	*p*-value	aOR (95% CI)*	*p*-value
	*N* = 153,440	*N* = 18,892				
Induction	63,544 (41.4%)	9,624 (50.9%)	1.45 (1.41, 1.50)	**< 0.001**	1.52 (1.47, 1.57)	**< 0.001**
**Mode of Delivery**						
Elective Caesarean Section	21,509 (14.0%)	3,825 (20.2%)	1.56 (1.50, 1.62)	**< 0.001**	1.38 (1.32, 1.44)	**< 0.001**
Emergency Caesarean Section	34,103 (22.2%)	5,032 (26.6%)	1.24 (1.20, 1.29)	**< 0.001**	1.16 (1.12, 1.20)	**< 0.001**
Spontaneous Vaginal Birth	76,338 (49.8%)	7,739 (40.9%)	0.74 (0.72, 0.77)	**< 0.001**	0.80 (0.77, 0.82)	**< 0.001**
Assisted Vaginal Birth	21,490 (14.0%)	2,296 (12.2%)	0.85 (0.81, 0.89)	**< 0.001**	0.90 (0.86, 0.94)	**< 0.001**
Preterm Delivery	8,019 (5.2%)	1,371 (7.3%)	1.40 (1.32, 1.49)	**< 0.001**	1.30 (1.22, 1.38)	**< 0.001**
Stillbirth	647 (0.4%)	48 (0.3%)	0.58 (0.43, 0.79)	**< 0.001**	0.50 (0.37, 0.68)	**< 0.001**
Polyhydramnios	1,286 (0.8%)	393 (2.1%)	2.50 (2.23, 2.81)	**< 0.001**	2.40 (2.13, 2.72)	**< 0.001**
PROM	22,743 (14.8%)	1,965 (10.4%)	0.67 (0.63, 0.70)	**< 0.001**	0.68 (0.65, 0.72)	**< 0.001**
GHTN	3,913 (2.6%)	619 (3.3%)	1.27 (1.17, 1.39)	**< 0.001**	1.23 (1.12, 1.35)	**< 0.001**
Pre-eclampsia	4,173 (2.7%)	660 (3.5%)	1.27 (1.17, 1.38)	**< 0.001**	1.18 (1.08, 1.28)	**< 0.001**
Eclampsia	78 (< 0.1%)	10 (< 0.1%)	1.04 (0.54, 2.01)	0.904	1.14 (0.58, 2.25)	0.709
Postpartum Haemorrhage	45,617 (29.7%)	5,944 (31.5%)	1.07 (1.04, 1.11)	**< 0.001**	1.06 (1.02, 1.09)	**< 0.001**

* Adjusted for age at delivery, ethnicity, IMD quintile, and family history of diabetes

Abbreviations: GDM = Gestational Diabetes Mellitus; PROM = Premature Rupture of Membranes; GHTN = Gestational Hypertension, OR = Odds Ratio; aOR = Adjusted Odds Ratio; CI = Confidence Interval

GDM was also associated with higher rates of preterm delivery (7.3% vs. 5.2%; aOR = 1.30, 95% CI 1.22–1.38) and polyhydramnios (2.1% vs. 0.8%; aOR = 2.40, 95% CI 2.13–2.72). Hypertensive complications were more prevalent in the GDM group, including GHTN (3.3% vs. 2.6%; aOR = 1.23, 95% CI 1.12–1.35) and pre-eclampsia (3.5% vs. 2.7%; aOR = 1.18, 95% CI 1.08–1.28), while no association was observed with eclampsia (< 0.1%; *p* = 0.707). PPH occurred at similar rates (31.5% vs. 29.7%) but remained independently associated with GDM after adjustment (aOR 1.06, 95% CI 1.02–1.09).

In contrast, GDM was associated with lower odds of PROM (aOR = 0.68, 95% CI 0.65–0.72) and stillbirth (0.3% vs. 0.4%; aOR = 0.50, 95% CI 0.37–0.68). Hospital stay was longer in the GDM group (3 days [IQR, 2–4] vs. 2 days [IQR, 1–3], *p* < 0.001), and spell costs were significantly higher (£4,674 [IQR, 3,942-6,471] vs. £3,971 [IQR, 3,204-5,175], *p* < 0.001).

Pregnancy outcomes also differed by GDM treatment modality (Additional file 2: Table S11). Insulin and combined insulin-metformin therapy were associated with higher rates of elective caesarean section and preterm delivery, whereas spontaneous vaginal birth was more frequent in the diet-only and metformin groups. PROM was most common in the diet-only group, and rates of postpartum haemorrhage increased incrementally with treatment intensity (all *p* < 0.05).

Interaction analyses for effect modification indicated that some associations were consistent across sociodemographic groups (Additional file 2: Table S12, Figures S2-4). No evidence of effect modification was observed by ethnicity or by IMD quintile for any outcome. Evidence of effect modification by maternal age was observed for induction of labour, all modes of delivery, PROM, and eclampsia (all adjusted interaction-*p* < 0.05), with no significant interactions detected for the remaining outcomes.

Stratified GEE models examining first-time GDM and ethnicity within BMI and deprivation strata are reported in Additional file 2: Tables S13 and S14. The associations between first-time GDM and pregnancy outcomes were directionally consistent across both BMI and IMD strata, with higher odds of induction, elective and emergency caesarean section, and polyhydramnios, and lower odds of spontaneous vaginal birth and PROM. For lower-incidence outcomes, significance was reached only in the low BMI analyses, including PTB (aOR 1.33, 95% CI 1.23–1.45 vs. 1.06, 95% CI 0.89–1.26 in high BMI), GHTN, and pre-eclampsia. Ethnic differences were broadly stable across deprivation strata but more variable by BMI, most notably for induction, where the elevated odds among Asian women relative to White women in the low BMI group (aOR 1.14, 95% CI 1.11–1.18) were not significant in the high BMI group (aOR 0.93, 95% CI 0.85–1.01), and for polyhydramnios, where White women had the highest odds in the high BMI stratum.

Compared with first-time GDM, recurrent GDM was associated with lower rates of induction of labour (43.3% vs. 51.9%), emergency caesarean section (18.4% vs. 27.7%), assisted vaginal birth (4.9% vs. 13.1%), PROM (6.4% vs. 10.9%), pre-eclampsia (1.9% versus 3.7%), and PPH (26.5% vs. 32.1%), whereas spontaneous vaginal birth (52.7% vs. 39.4%) and elective caesarean section (24.0% vs. 19.8%) were more frequent (all *p* < 0.001). Rates of PTB, stillbirth, polyhydramnios, GHTN, and eclampsia did not differ significantly (Additional file 2: Table S12).

## Discussion

### Summary of findings

The overall incidence of GDM in NWL was 109.6 per 1,000 births, with 10.9% of all pregnancies being affected. GDM incidence demonstrated a substantial increase over the nine-year period, rising from 80.5 per 1,000 births in 2017 to 138 per 1,000 births in 2025, reaching levels comparable with recent global estimates. This marked upward trend occurred despite a slight decline in overall annual births, suggesting genuine increases in incidence rather than demographic shifts alone.

Asian women demonstrated nearly three-fold higher odds of GDM compared to their White counterparts (OR = 2.73, 95% CI 2.63–2.84), and risk increased with greater maternal age, higher BMI, prior miscarriage, and a family history of diabetes. An inverse association with smoking was observed, likely explained by underlying demographic differences. Smokers were slightly younger, had lower BMIs, and were more likely to be of White ethnicity compared with non-smokers (69% vs. 33%), with a lower proportion of Asian ethnicity as well (8.4% vs. 40%).

Clear socioeconomic inequalities emerged across deprivation quintiles, with women in the most deprived quintile experiencing almost 40% higher GDM incidence than those in the least deprived (119 vs. 85.4 per 1,000 births). Furthermore, substantial geographic variation was also observed, with certain boroughs such as Harrow, Brent, Ealing, and Hounslow exhibiting almost two-fold incidence rates, compared to Kensington & Chelsea, the most expensive borough in London and the UK. The boroughs with higher incidence are characterised by higher levels of socioeconomic deprivation, as reflected by lower IMD quintiles. These patterns are therefore likely to reflect differences in population composition and area-level socioeconomic conditions, rather than individual behaviours in isolation [20, 21].

GDM was associated with different rates of obstetric outcomes after adjusting for age, ethnicity, IMD quintile, and family history of diabetes. Women with GDM had higher rates of labour induction (51% vs. 41%), operative delivery (both elective and emergency caesarean sections), and preterm birth (7.3% vs. 5.2%). Lower rates of stillbirth and PROM were observed. GDM pregnancies also had higher rates of induction, preterm birth, and incurred increased healthcare costs (median £4,674 vs. £3,971) and longer hospital stays (median 3 vs. 2 days).

### Comparison with published literature

The incidence observed in this NWL cohort substantially exceeds national estimates. The Royal College of Obstetricians and Gynaecologistsestimates that 4–5 in every 100 pregnant women are affected, and NHS England reports comparable figures with approximately 30,000 of 700,000 annual births affected [22, 23], corresponding to an incidence rate of up to 50 per 1,000 births. This is less than half the incidence observed in this study’s cohort. An earlier analysis of births in NWL maternity units between 1988 and 2000 reported a prevalence of below 1% [24]; and two UK Biobank studies reported prevalence at 0.63% [25] and 0.55% [26]. Compared to previous studies, the prevalence observed was substantially higher at 10.9% highlighting a marked temporal increase in the burden of GDM across the region.

The risk factor profile is consistent with existing literature [9]. The observed increase in the odds of GDM with advancing maternal age (aOR = 1.08, 95% CI: 1.08–1.09) is consistent with findings from a previous study within an NWL Trust (2016–2019), which reported a comparable adjusted effect (age aOR = 1.076) after accounting for BMI, ethnicity, and IMD [27]. That study also identified higher BMI and non-White ethnicity as risk factors for GDM incidence. However, in contrast to the present findings, they did not find any significant association with IMD.

The lower rates of stillbirth and PROM observed were unexpected, given that GDM has been reported to increase the risk of both [28, 29]. A potential explanation is the intensive clinical management GDM pregnancies receive due to their high-risk nature: higher rates of induction, elective delivery, and closer antenatal surveillance may collectively limit progression to high-risk late gestational ages, rather than GDM itself being protective against stillbirth. This is consistent with a recent systematic review from over 92 million women, which found that timely diagnosis and effective management strategies such as induction of labour and increased antenatal care in high-income countries may explain the reduced risk of stillbirth in women with GDM [30]. Similarly, earlier planned deliveries, such as elective caesareans and labour induction, may be artificially lowering the observed PROM rates in the GDM cohort.

The longer hospital stay observed in GDM pregnancies may be attributed to higher rates of labour induction and caesarean delivery, as also reported in previous studies [31, 32]. Although there is no national recommendation for a routine observation period following birth [33], capillary blood glucose monitoring is performed routinely during admission, and persistently elevated readings may necessitate prolonged stays. Additionally, newborn monitoring requirements and potential neonatal unit admission for complications such as neonatal hypoglycaemia may contribute, though these are not captured in the present dataset.

Data quality due to coding is known to be a problem in the UK, as the national Maternity Services Dataset has been described as ‘immature’ with ‘suboptimal’ data quality when it comes to GDM, which may affect reported incidence [34]. The higher incidence observed in the present NWL cohort is likely attributed to its ethnically and socio-demographically diverse population, which is associated with an inherently increased risk of GDM development [27, 35].

The distribution of GDM cases by IMD was consistent with published findings from the NHS GDM Audit 2024-25, however, notable differences were observed in ethnic composition. Nationally, over half of the women were White (59%), compared to 23% in this NWL cohort [36]. This delta likely reflects regional demographic variation, as London has the lowest proportion of White people compared to other regions in England [37]. Mode of delivery was predominantly by caesarean section, accounting for 44.3% deliveries in England in 2024-25, which is comparable to this NWL cohort, where the rate was 47% across the whole period [36].

### Strengths and limitations

This study utilised a large population-based dataset with comprehensive coverage across diverse ethnic, sociodemographic, and clinical groups. Such diversity is not always reflected in other commonly used UK datasets, such as the UK Biobank, which is approximately 95% White [38]. The nature of the diverse population enhances both representativeness and generalisability of findings beyond NWL. In contrast to previous UK-based studies, reporting lower GDM rates, the prevalence in this cohort was over 10%, minimising bias associated with small sample sizes and event numbers, thus increasing statistical power. The availability of nearly a decade of data enabled robust temporal analyses, allowing assessment of whether observed increases in GDM incidence were driven by changes in overall delivery volumes or by shifts in proportional incidence. Furthermore, the WSIC database allows for linkage between primary and secondary care records, thus providing near-complete NHS provider coverage across NWL, a database previously used for GDM research [39].

Several limitations should be acknowledged. Firstly, the study is subject to potential coding inconsistencies across multiple provider organisations, spanning four NHS Trusts. As with all studies utilising HES data, findings are dependent on the accuracy and completeness of diagnostic and procedural coding, as well as on the extent of missing data [40, 41]. For example, in this cohort, around 1,300 births were found to have conflicting delivery method coding, i.e., both vaginal and caesarean section or both an elective and emergency caesarean; those records were excluded from the analysis. In addition, misclassification of certain obstetric outcomes may have occurred; for instance, codes used for PROM may sometimes be applied interchangeably with PPROM, potentially introducing inconsistencies in outcome classification. Furthermore, incomplete data were particularly evident for smoking status and BMI, with the latter having missing values in approximately 28% of records, a limitation commonly reported in HES-based analyses [42, 43]. Importantly, neonatal outcomes commonly associated with GDM, such as neonatal hypoglycaemia, jaundice, or admission to neonatal intensive care units, were not available, which limited the ability to assess the full impact on neonatal morbidity. Regarding GDM diagnosis, England operates a risk-factor-based rather than universal screening programme, and the NICE NG3 diagnostic criteria applied throughout the study period have remained consistent, predating the most substantial threshold changes introduced following the IADPSG revision [44]. The observed trends therefore reflect variation within a single diagnostic framework, though residual bias attributable to improvements in screening uptake or coding completeness over time cannot be fully excluded. Finally, population mobility represents an additional challenge, as a substantial proportion of patients may move into or out of London and across regions over time. However, restricting the analysis to women both residing in and giving birth within NWL ensured that the findings accurately reflect the population served in the region.

### Future research and policy implications

Future research should prioritise the investigation of long-term maternal and offspring outcomes, including metabolic health, through linked mother-baby datasets. Cost-effectiveness analyses of alternative GDM screening strategies are warranted, alongside qualitative research exploring barriers to diagnosis, engagement with care, and continuity of follow-up. Further work should also examine provider-level variation in coding practices and clinical management, as well as how care is delivered and adapted for women from ethnically diverse backgrounds and for those whose primary language is not English, groups that constitute a substantial proportion of GDM-affected pregnancies in NWL and globally.

Understanding disparities in access to care and clinical outcomes is essential to disentangle the relative contributions of individual risk factors, such as genetic susceptibility or obesity, from structural and contextual determinants of GDM risk and management. Reducing health inequalities is a major priority as outlined in the NHS 10-year Long Term Plan [45]. While ethnicity and advanced maternal age are non-modifiable, the findings highlight obesity and deprivation as actionable targets for prevention. Obesity disproportionately affects women in more deprived areas [46], and these factors are not independent. Local commissioners should therefore extend beyond screening pathways to equity-focused prevention addressing the structural determinants. Given that antenatal diabetes clinics are already operating above capacity [47], there is a clear need for health equity-focused prevention strategies and earlier intervention, spanning pre-conception counselling, culturally appropriate lifestyle support, and early risk stratification, to help mitigate the rising GDM prevalence. Targeting obesity reduction in high-deprivation areas is the most actionable option available in reducing incidence and improving maternal and offspring health outcomes.

## Conclusions

To the authors' knowledge, this study represents the largest, most up-to-date population-based analysis of GDM epidemiology in NWL. The findings underscore the substantial and persistent socioeconomic and geographic inequalities in GDM burden, highlighting the need for targeted, culturally responsive prevention and care strategies. By providing robust, real-world evidence from a diverse urban population, this study offers important insights for public health planning and clinical practice. The findings suggest that compared with the NICE approach, a place-based approach may be more appropriate, incorporating local risk factors, developing tailored prediction models, or, in areas of high prevalence, considering universal screening.

## Supplementary Information

Below is the link to the electronic supplementary material.

Additional File 1: STROBE Checklist

Additional File 2: Tables S1–S15 and Figures S1–S4

## Data Availability

The data that support the findings of this study are available from Discover-NOW but restrictions apply to the availability of these data, which were used under license for the current study, and so are not publicly available. Researchers may access the raw data by submitting an application to Discover-NOW’s Data Access Committee (DAC) and obtaining the necessary approvals.

## Abbreviations

aOR: Adjusted Odds Ratio
BMI: Body Mass Index
CI: Confidence Interval
GDM: Gestational Diabetes Mellitus
GEE: Generalised Estimating Equations
GHTN: Gestational Hypertension
GP: General Practice
HES: Hospital Episode Statistics
IADPSG: International Association of Diabetes and Pregnancy Study Groups
ICD: International Classification of Diseases
IMD: Index of Multiple Deprivation
IQR: Interquartile Range
NHS: National Health Service
NICE: National Institute for Health and Care Excellence
NWL: North West London
OPCS: Office of Population Censuses and Surveys Classification
OR: Odds Ratio
PAF: Population Attributable Fraction
PPH: Postpartum Haemorrhage
PPROM: Preterm Premature Rupture of Membranes
PROM: Premature Rupture of Membranes
PTB: Preterm Birth
SD: Standard Deviation
STROBE: STrengthening the Reporting of OBservational studies in Epidemiology
WSIC: Whole Systems Integrated Care
