# Supplementary material for: Trends, disparities, and pregnancy outcomes of gestational diabetes mellitus in North West London: A nine-year population-based cohort study
Source: BMC Med. 2026 Jul 28;24:586. doi: 10.1186/s12916-026-05103-4 (PMC13693047; doi:10.1186/s12916-026-05103-4)
Supplement: Supplementary file 2 [file 12916_2026_5103_MOESM2_ESM.docx]

**Trends, Disparities, and Pregnancy Outcomes of Gestational Diabetes Mellitus in North West London: A Nine-year Population-based Cohort Study**

[Table S1 – Clinical Pregnancy Outcomes	2](#_Toc236054165)

[Table S2 – Diagnosis CodesTable S2 – Obstetric Outcomes Diagnosis Codes (ICD-10)	3](#_Toc236054166)

[Table S3 – Smoking SNOMED CT Codes	4](#_Toc236054167)

[Table S4 – Family History of Diabetes SNOMED CT Codes	5](#_Toc236054168)

[Table S5 – BMI SNOMED CT Codes	5](#_Toc236054169)

[Table S6 – Miscarriage SNOMED CT Codes	5](#_Toc236054170)

[Table S7 – North West London Hospital Organisation Codes	6](#_Toc236054171)

[Table S8 – Patient Characteristic by GDM Treatment Modality	7](#_Toc236054172)

[Table S9 – Unadjusted Population Attributable Fractions for GDM Stratified by Ethnicity, BMI, IMD, and Maternal Age	8](#_Toc236054173)

[Table S10 – Distribution of Characteristics by Smoking Status	9](#_Toc236054174)

[Table S11 – Obstetric Outcomes by GDM Treatment Modality	9](#_Toc236054175)

[Table S12 – Tests for effect modification between GDM and pregnancy outcomes	10](#_Toc236054176)

[Table S13 –- GEE-Estimated Associations Between First-time GDM, Ethnicity, and Obstetric Outcomes, Stratified by Body Mass Index	11](#_Toc236054177)

[Table S14 –- GEE-Estimated Associations Between First-time GDM, Ethnicity, and Obstetric Outcomes, Stratified by Deprivation Index (IMD)	14](#_Toc236054178)

[Table S15 – Obstetric Outcomes by first-time GDM vs recurrent-GDM pregnancy	17](#_Toc236054179)

[Figure S1 -– Cohort derivation flow diagram	18](#_Toc236054180)

[Figure S2 – Prevalence (%) of outcomes by Age group and GDM status	19](#_Toc236054181)

[Figure S3 – Prevalence (%) of outcomes by Ethnicity and GDM status	19](#_Toc236054182)

[Figure S4 – Prevalence (%) of outcomes by IMD quintile and GDM status	20](#_Toc236054183)

**Supplementary Material**

# Table S1 – Clinical Pregnancy Outcomes

| **Outcome** | **OPCS-4 Code** | **Code Description** |
| --- | --- | --- |
| Induction | R14 | Surgical induction of labour |
|  | R14.1 | Forewater rupture of amniotic membrane |
|  | R14.2 | Hindwater rupture of amniotic membrane |
|  | R14.8 | Other specified |
|  | R14.9 | Unspecified |
|  | R15 | Other induction of labour (includes other augmentation of labour) |
|  | R15.1 | Medical induction of labour (prostaglandins, oxytocin, misoprostol) |
|  | R15.2 | Mechanical induction of labour (osmotic dilator, balloon dilator) |
|  | R15.8 | Other specified induction of labour |
|  | R15.9 | Unspecified induction of labour |
| Elective Caesarean Section | R17 | Elective caesarean section |
|  | R17.1 | Elective upper uterine segment caesarean delivery |
|  | R17.2 | Elective lower uterine segment caesarean delivery |
|  | R17.8 | Other specified elective caesarean delivery |
|  | R17.9 | Unspecified elective caesarean delivery |
| Emergency Caesarean Section | R18 | Emergency caesarean section |
|  | R18.1 | Upper uterine segment caesarean delivery NEC |
|  | R18.2 | Lower uterine segment caesarean delivery NEC |
|  | R18.8 | Other specified caesarean delivery |
|  | R18.9 | Unspecified caesarean delivery |
| Spontaneous Vaginal Birth | R20.1 | Spontaneous breech delivery |
|  | R24 | Normal delivery |
|  | R24.9 | Normal delivery – all |
| Assisted Vaginal Birth | R19 | Breech extraction delivery |
|  | R19.1 | Breech extraction delivery with version |
|  | R19.8 | Other specified breech extraction delivery |
|  | R19.9 | Unspecified breech extraction delivery |
|  | R20 | Other breech delivery |
|  | R20.2 | Assisted breech delivery |
|  | R20.8 | Other specified breech delivery |
|  | R20.9 | Unspecified breech delivery |
|  | R21 | Forceps cephalic delivery |
|  | R21.1 | High forceps cephalic delivery with rotation |
|  | R21.2 | High forceps cephalic delivery NEC |
|  | R21.3 | Mid forceps cephalic delivery with rotation |
|  | R21.4 | Mid forceps cephalic delivery NEC |
|  | R21.5 | Low forceps cephalic delivery |
|  | R21.8 | Other specified forceps cephalic delivery |
|  | R21.9 | Unspecified forceps cephalic delivery |
|  | R22 | Vacuum delivery |
|  | R22.1 | High vacuum delivery |
|  | R22.2 | Low vacuum delivery |
|  | R22.3 | Vacuum delivery before full dilation of cervix |
|  | R22.8 | Other specified vacuum delivery |
|  | R22.9 | Unspecified vacuum delivery |
|  | R23 | Cephalic vaginal delivery with abnormal presentation of head at delivery without instrument |
|  | R23.1 | Manipulative cephalic vaginal delivery with abnormal presentation without instrument |
|  | R23.2 | Non-manipulative cephalic vaginal delivery with abnormal presentation without instrument |
|  | R23.8 | Other specified abnormal cephalic vaginal delivery |
|  | R23.9 | Unspecified abnormal cephalic vaginal delivery |

# Table S2 – Diagnosis CodesTable S2 – Obstetric Outcomes Diagnosis Codes (ICD-10)

| **Outcome** | **ICD-10 Code** | **Code Description** |
| --- | --- | --- |
| GDM | O24.4 | Gestational diabetes mellitus |
| Preterm Delivery | O60 | Preterm labour and delivery |
|  | O60.0 | Preterm labour without delivery |
|  | O60.1 | Preterm spontaneous labour with preterm delivery |
|  | O60.2 | Preterm spontaneous labour with term delivery |
|  | O60.3 | Preterm delivery without spontaneous labour |
|  | P07.2 | Extreme immaturity |
|  | P07.3 | Other preterm infants |
|  | P59.0 | Neonatal jaundice associated with preterm delivery |
|  | P61.2 | Anaemia of prematurity |
| Stillbirth | Z37.1 | Single stillbirth |
|  | Z37.4 | Twins, both stillborn |
|  | Z37.7 | Other multiple births, all stillborn |
| Polyhydramnios | O40 | Polyhydramnios |
| Premature Rupture of Membranes (PROM) | O42 | Premature rupture of membranes |
|  | O42.0 | Premature rupture of membranes, onset of labour within 24 hours |
|  | O42.1 | Premature rupture of membranes, onset of labour after 24 hours |
|  | O42.8 | Premature rupture of membranes, labour delayed by therapy |
|  | O42.9 | Premature rupture of membranes, unspecified |
| Gestational Hypertension | O13 | Gestational hypertension |
| Pre-eclampsia | O14 | Pre-eclampsia |
|  | O14.0 | Mild to moderate pre-eclampsia |
|  | O14.1 | Severe pre-eclampsia |
|  | O14.2 | HELLP syndrome |
|  | O14.9 | Pre-eclampsia, unspecified |
| Eclampsia | O15 | Eclampsia |
|  | O15.0 | Eclampsia in pregnancy |
|  | O15.1 | Eclampsia in labour |
|  | O15.2 | Eclampsia in the puerperium |
|  | O15.9 | Eclampsia, unspecified as to time period |
| Postpartum Haemorrhage | O72 | Postpartum haemorrhage |
|  | O72.0 | Third-stage haemorrhage |
|  | O72.1 | Other immediate postpartum haemorrhage |
|  | O72.2 | Delayed and secondary postpartum haemorrhage |
|  | O72.9 | Postpartum coagulation defects |

# Table S3 – Smoking SNOMED CT Codes

| **Smoking Category** | **SNOMED Code** | **SNOMED Term** |
| --- | --- | --- |
| Ex-smoker | 8517006 | Ex-smoker |
| Ex-smoker | 53896009 | Tolerant ex-smoker |
| Ex-smoker | 160617001 | Stopped smoking |
| Ex-smoker | 160620009 | Ex-pipe smoker |
| Ex-smoker | 160621008 | Ex-cigar smoker |
| Ex-smoker | 160625004 | Date ceased smoking |
| Ex-smoker | 228486009 | Time since stopped smoking |
| Ex-smoker | 266921000 | Ex-trivial cigarette smoker (<1/day) |
| Ex-smoker | 266922007 | Ex-light cigarette smoker (1-9/day) |
| Ex-smoker | 266923002 | Ex-moderate cigarette smoker (1-9/day) |
| Ex-smoker | 266924008 | Ex-heavy cigarette smoker (20-39/day) |
| Ex-smoker | 266925009 | Ex-very heavy cigarette smoker (40+/day) |
| Ex-smoker | 266928006 | Ex-cigarette amount unknown |
| Ex-smoker | 281018007 | Ex-cigarette smoker |
| Ex-smoker | 360890004 | Intolerant ex-smoker |
| Ex-smoker | 360900008 | Aggressive ex-smoker |
| Ex-smoker | 735128000 | Ex-smoker for less than 1 year |
| Ex-smoker | 48031000119106 | Ex-smoker for more than 1 year |
| Ex-smoker | 109203100000108 | Ex-smoker amount unknown |
| Ex-smoker | 109204100000104 | Ex-very heavy smoker (40+/day) |
| Ex-smoker | 109207100000105 | Ex-heavy smoker (20-39/day) |
| Ex-smoker | 109209100000109 | Ex-moderate smoker (10-19/day) |
| Ex-smoker | 109211100000104 | Ex-light smoker (1-9/day) |
| Ex-smoker | 109213100000107 | Ex-trivial smoker (<1/day) |
| Ex-smoker | 492191000000103 | Ex roll-up cigarette smoker |
| Ex-smoker | 517211000000106 | Recently stopped smoking |
| Non-smoker | 8392000 | Non-smoker |
| Non-smoker | 160618006 | Current non-smoker |
| Non-smoker | 266919005 | Never smoked tobacco |
| Non-smoker | 221000119102 | Never smoked any substance |
| Non-smoker | 505681000000109 | Non-smoker annual review |
| Smoker | 56578002 | Moderate smoker (20 or less per day) |
| Smoker | 56771006 | Heavy smoker (over 20 per day) |
| Smoker | 59978006 | Cigar smoker |
| Smoker | 65568007 | Cigarette smoker |
| Smoker | 77176002 | Smoker |
| Smoker | 82302008 | Pipe smoker |
| Smoker | 134406006 | Smoking reduced |
| Smoker | 160603005 | Light cigarette smoker (1-9 cigs/day) |
| Smoker | 160604004 | Moderate cigarette smoker (10-19/day) |
| Smoker | 160605003 | Heavy cigarette smoker (20-39 cigs/day) |
| Smoker | 160606002 | Very heavy cigarette smoker (40+/day) |
| Smoker | 160612007 | Keeps trying to stop smoking |
| Smoker | 160613002 | Admitted tobacco consumption per day |
| Smoker | 160616005 | Trying to give up smoking |
| Smoker | 160619003 | Rolls own cigarettes |
| Smoker | 225934006 | Smokes in bed |
| Smoker | 230056004 | Cigarette consumption |
| Smoker | 230057008 | Cigar consumption |
| Smoker | 230058003 | Pipe tobacco consumption |
| Smoker | 230059006 | Occasional cigarette smoker |
| Smoker | 230060001 | Light cigarette smoker |
| Smoker | 230062009 | Moderate cigarette smoker |
| Smoker | 230063004 | Heavy cigarette smoker |
| Smoker | 230064005 | Very heavy cigarette smoker |
| Smoker | 230065006 | Chain smoker |
| Smoker | 266918002 | Tobacco smoking consumption |
| Smoker | 266920004 | Trivial cigarette smoker (less than 1/day) |
| Smoker | 266927001 | Tobacco smoking consumption unknown |
| Smoker | 266929003 | Smoking started |
| Smoker | 308438006 | Smoking restarted |
| Smoker | 365981007 | Tobacco smoking behaviour - finding |
| Smoker | 394871007 | Thinking about stopping smoking |
| Smoker | 394873005 | Not interested in stopping smoking |
| Smoker | 401159003 | Reason for restarting smoking |
| Smoker | 401201003 | Cigarette pack-years |
| Smoker | 413173009 | Minutes from waking to first tobacco use |
| Smoker | 446172000 | Failed attempt to stop smoking |
| Smoker | 449868002 | Smokes tobacco daily |
| Smoker | 782516008 | Number of calculated smoking packs per year |
| Smoker | 109248100000104 | Number of calculated smoking pack-years |
| Smoker | 203191000000107 | Wants to stop smoking |
| Smoker | 428041000124106 | Occasional tobacco smoker |
| Smoker | 836001000000109 | Waterpipe tobacco consumption |

# Table S4 – Family History of Diabetes SNOMED CT Codes

| **SNOMED Code** | **SNOMED Term** |
| --- | --- |
| 430679000 | Family history of diabetes mellitus type 2 |
| 160303001 | Family history of diabetes mellitus |
| 430678008 | Family history of diabetes mellitus type 1 |

# Table S5 – BMI SNOMED CT Codes

| **SNOMED CT Code** | **SNOMED Term** |
| --- | --- |
| 60621009 | Body mass index |
| 301331008 | Finding of body mass index |

# Table S6 – Miscarriage SNOMED CT Codes

| SNOMED CT Code | SNOMED Term |
| --- | --- |
| 2781009 | Miscarriage complicated by delayed and/or excessive haemorrhage |
| 16607004 | Missed miscarriage |
| 17369002 | Miscarriage |
| 19169002 | Miscarriage in first trimester |
| 23793007 | Miscarriage without complication |
| 30806007 | Miscarriage with endometritis |
| 34614007 | Miscarriage with complication |
| 54048003 | Threatened miscarriage |
| 58990004 | Miscarriage complicated by damage to pelvic organs and/or tissues |
| 59363009 | Inevitable miscarriage |
| 62688006 | Surgical treatment of miscarriage of any trimester |
| 67465009 | Miscarriage with sepsis |
| 73790007 | Threatened miscarriage in first trimester |
| 85116003 | Miscarriage in second trimester |
| 156072005 | Incomplete miscarriage |
| 156073000 | Complete miscarriage |
| 184339009 | Miscarriage at 8 to 28 weeks |
| 198644001 | Incomplete miscarriage with genital tract or pelvic infection |
| 198645000 | Incomplete miscarriage with delayed or excessive haemorrhage |
| 198646004 | Incomplete miscarriage with damage to pelvic organs or tissues |
| 198655001 | Complete miscarriage with genital tract or pelvic infection |
| 198656000 | Complete miscarriage with delayed or excessive haemorrhage |
| 198657009 | Complete miscarriage with damage to pelvic organs or tissues |
| 198661003 | Complete miscarriage with shock |
| 275421004 | Miscarriage with heavy bleeding |
| 307733001 | Inevitable miscarriage complete |
| 307734007 | Complete inevitable miscarriage complicated by genital tract and pelvic infection |
| 307735008 | Complete inevitable miscarriage complicated by delayed or excessive haemorrhage |
| 307737000 | Inevitable miscarriage incomplete |
| 307738005 | Complete inevitable miscarriage without complication |
| 307748007 | Incomplete inevitable miscarriage without complication |
| 307749004 | Incomplete inevitable miscarriage complicated by genital tract and pelvic infection |
| 307750004 | Complete inevitable miscarriage complicated by embolism |
| 307752007 | Incomplete inevitable miscarriage complicated by delayed or excessive haemorrhage |
| 363581007 | Pregnancy with abortive outcome |
| 413338003 | Incomplete miscarriage with complication |
| 609525000 | Miscarriage of tubal ectopic pregnancy |

# Table S7 – North West London Hospital Organisation Codes

| **Organisation Code** | **NHS Trust** |
| --- | --- |
| RQM | Chelsea and Westminster Hospital NHS Foundation Trust |
| RYS | Imperial College Healthcare NHS Trust |
| R1K | London North West University Healthcare NHS Trust |
| RAS | The Hillingdon Hospital NHS Foundation Trust |

# Table S8 – Patient Characteristic by GDM Treatment Modality

|  | **Diet N=13,421** | **Metformin N=2,833** | **Insulin N=1,117** | **Combined N=1,521** | **p-value** |
| --- | --- | --- | --- | --- | --- |
| **Sociodemographic Characteristics** | | | | | |
| **Age at Delivery (years)** | 33.5 (5.1) | 33.9 (5.0) | 34.4 (5.0) | 34.5 (5.0) | **<0.001** |
| **Ethnicity** |  |  |  |  | **<0.001** |
| Asian | 6,003 (45%) | 1,739 (61%) | 524 (47%) | 980 (64%) |  |
| Black | 1,514 (11%) | 238 (8.4%) | 140 (13%) | 109 (7.2%) |  |
| Mixed | 444 (3.3%) | 72 (2.5%) | 42 (3.8%) | 36 (2.4%) |  |
| Not Recorded | <5 (<0.1%) | <5 (<0.1%) | 0 (0%) | 0 (0%) |  |
| Other | 1,955 (15%) | 360 (13%) | 161 (14%) | 184 (12%) |  |
| White | 3,502 (26%) | 423 (15%) | 250 (22%) | 212 (14%) |  |
| **First Language** |  |  |  |  | **<0.001** |
| English | 4,430 (33%) | 812 (29%) | 373 (33%) | 431 (28%) |  |
| Not English | 6,345 (47%) | 1,514 (53%) | 503 (45%) | 801 (53%) |  |
| Not Recorded | 2,646 (20%) | 507 (18%) | 241 (22%) | 289 (19%) |  |
| **IMD 2019** |  |  |  |  | **<0.001** |
| 1 (Most deprived) | 2,051 (15%) | 344 (12%) | 196 (18%) | 193 (13%) |  |
| 2 | 4,955 (37%) | 1,084 (38%) | 423 (38%) | 634 (42%) |  |
| 3 | 3,878 (29%) | 835 (29%) | 326 (29%) | 445 (29%) |  |
| 4 | 1,909 (14%) | 426 (15%) | 130 (12%) | 174 (11%) |  |
| 5 (Least deprived) | 628 (4.7%) | 144 (5.1%) | 42 (3.8%) | 75 (4.9%) |  |
| **Borough** |  |  |  |  | **<0.001** |
| Brent | 2,384 (18%) | 649 (23%) | 135 (12%) | 274 (18%) |  |
| Ealing | 2,772 (21%) | 546 (19%) | 310 (28%) | 359 (24%) |  |
| Hammersmith and Fulham | 915 (6.8%) | 83 (2.9%) | 82 (7.3%) | 37 (2.4%) |  |
| Harrow | 1,613 (12%) | 591 (21%) | 61 (5.5%) | 201 (13%) |  |
| Hillingdon | 2,194 (16%) | 487 (17%) | 131 (12%) | 356 (23%) |  |
| Hounslow | 2,187 (16%) | 360 (13%) | 319 (29%) | 249 (16%) |  |
| Kensington and Chelsea | 512 (3.8%) | 45 (1.6%) | 27 (2.4%) | 22 (1.4%) |  |
| Westminster | 844 (6.3%) | 72 (2.5%) | 52 (4.7%) | 23 (1.5%) |  |
| **Clinical Characteristics** | | | | | |
| **BMI (kg/m^2^)** |  |  |  |  | **<0.001** |
| <18.5 | 1,083 (8.1%) | 177 (6.2%) | 64 (5.7%) | 59 (3.9%) |  |
| 18.5-25 | 5,946 (44%) | 1,134 (40%) | 428 (38%) | 486 (32%) |  |
| 25-30 | 800 (6.0%) | 200 (7.1%) | 79 (7.1%) | 108 (7.1%) |  |
| 30+ | 1,757 (13%) | 482 (17%) | 245 (22%) | 424 (28%) |  |
| Missing | 3,835 (29%) | 840 (30%) | 301 (27%) | 444 (29%) |  |
| **Smoking Status** |  |  |  |  | **<0.001** |
| Ex-smoker | 884 (6.6%) | 122 (4.3%) | 79 (7.1%) | 90 (5.9%) |  |
| Non-smoker | 10,808 (81%) | 2,461 (87%) | 902 (81%) | 1,304 (86%) |  |
| Smoker | 924 (6.9%) | 152 (5.4%) | 102 (9.1%) | 87 (5.7%) |  |
| Unknown | 805 (6.0%) | 98 (3.5%) | 34 (3.0%) | 40 (2.6%) |  |
| **Family History of Diabetes** | 6,491 (48%) | 1,609 (57%) | 657 (59%) | 939 (62%) | **<0.001** |
| **History of Miscarriage** | 2,077 (15%) | 511 (18%) | 245 (22%) | 285 (19%) | **<0.001** |
| *n(%) or Mean (SD); Wilcoxon Rank Sum Test; Pearson Chi-Square Test* | | | | | |
| Abbreviations: GDM = Gestational Diabetes Mellitus; BMI = Body Mass Index; IMD = Index of Multiple Deprivation | | | | | |

# Table S9 – Unadjusted Population Attributable Fractions for GDM Stratified by Ethnicity, BMI, IMD, and Maternal Age

| **Stratum** | **Category** | **Unadjusted PAF (%) (95% CI)** |
| --- | --- | --- |
| **Ethnicity** | | |
| Ethnicity | Asian | 22.63 (22.19, 23.07) |
| Ethnicity | Black | 0.87 (0.80, 0.95) |
| Ethnicity | Mixed | -0.61 (-0.63, -0.59) |
| Ethnicity | Other | -10.12 (-12.59, -7.67) |
| Ethnicity | White | 41.42 (40.53, 42.32) |
| Ethnicity | Not Recorded | 20.68 (-87.13, 77.62) |
| **Body Mass Index** | | |
| BMI | <30 kg/m² | 11.00 (10.81, 11.20) |
| BMI | ≥30 kg/m² | -81.53 (-84.56, -78.49) |
| **Index of Multiple Deprivation** | | |
| IMD | Quintile 1-2 | 7.21 (6.64, 7.78) |
| IMD | Quintile 3-5 | 2.61 (2.17, 3.06) |
| **Maternal Age at Delivery (years)** | | |
| Age | <20 | -0.90 (-0.90, -0.89) |
| Age | 20-24 | -5.96 (-5.99, -5.94) |
| Age | 25-29 | -7.69 (-7.84, -7.54) |
| Age | 30-34 | 0.16 (-0.17, 0.48) |
| Age | 35-39 | 9.44 (9.18, 9.69) |
| Age | 40-44 | 4.59 (4.52, 4.67) |
| Age | ≥45 | 0.44 (0.43, 0.45) |

Abbreviations: PAF = Population Attributable Fraction; CI = Confidence Interval; BMI = Body Mass Index; IMD = Index of Multiple Deprivation.

Population attributable fractions represent the proportional reduction in GDM cases that would occur if the exposure category were set to the reference level, assuming a causal relationship. Negative values indicate strata associated with a lower GDM burden relative to the reference category and do not imply a protective causal effect; they reflect the combination of category-specific relative risk and exposure prevalence used in PAF calculations.

# Table S10 – Distribution of Characteristics by Smoking Status

|  | **Non-smoker**  **(N = 128,830)** | **Ex-smoker**  **(N = 13,330)** | **Smoker**  **(N = 15,982)** | **p-value** |
| --- | --- | --- | --- | --- |
| **Maternal age at delivery, years** | 31.9 (5.3) | 33.1 (5.4) | 31.3 (5.7) | **<0.001** |
| **BMI category (kg/m^2^)** |  |  |  | **<0.001** |
| <18.5 | 14,264 (11.1%) | 1,393 (10.5%) | 2,237 (14.0%) |  |
| 18.5–25 | 64,554 (50.1%) | 7,457 (55.9%) | 8,423 (52.7%) |  |
| 25–30 | 6,606 (6.8%) | 585 (4.4%) | 726 (4.5%) |  |
| ≥30 | 11,232 (5.2%) | 1,133 (8.5%) | 1,406 (8.8%) |  |
| Missing | 32,174 (25.0%) | 2,762 (20.7%) | 3,190 (20.0%) |  |
| **Ethnicity** |  |  |  | **<0.001** |
| Asian | 52,174 (40.5%) | 1,290 (9.7%) | 1,335 (8.4%) |  |
| Black | 13,207 (10.3%) | 862 (6.5%) | 1,329 (8.3%) |  |
| Mixed | 4,471 (3.5%) | 655 (4.9%) | 745 (4.7%) |  |
| Not recorded | 29 (<0.1%) | 0 (0%) | <5 (<0.1%) |  |
| Other | 16,816 (13.1%) | 1,360 (10.2%) | 1,474 (9.2%) |  |
| White | 42,133 (32.7%) | 9,163 (68.8%) | 11,096 (69.4%) |  |

# Table S11 – Obstetric Outcomes by GDM Treatment Modality

|  | **Diet N=13,421** | **Metformin N=2,833** | **Insulin N=1,117** | **Combined N=1,521** | **p-value*** |
| --- | --- | --- | --- | --- | --- |
| **Induction** | 6,751 (50.3%) | 1,479 (52.2%) | 593 (53.1%) | 801 (52.7%) | **0.049** |
| **Mode of Delivery** |  |  |  |  |  |
| Elective Caesarean Section | 2,525 (18.8%) | 562 (19.8%) | 316 (28.3%) | 422 (27.7%) | **<0.001** |
| Emergency Caesarean Section | 3,541 (26.4%) | 762 (26.9%) | 288 (25.8%) | 441 (29.0%) | 0.152 |
| Spontaneous Vaginal Birth | 5,685 (42.4%) | 1,166 (41.2%) | 380 (34.0%) | 508 (33.4%) | **<0.001** |
| Assisted Vaginal Birth | 1,670 (12.4%) | 343 (12.1%) | 133 (11.9%) | 150 (9.9%) | **0.035** |
| **Preterm Delivery** | 951 (7.1%) | 196 (6.9%) | 101 (9.0%) | 123 (8.1%) | **0.048** |
| **Stillbirth** | 41 (0.3%) | <5 (0.1%) | <5 (0.2%) | <5 (0.1%) | 0.207 |
| **Polyhydramnios** | 277 (2.1%) | 51 (1.8%) | 33 (3.0%) | 32 (2.1%) | 0.151 |
| **PROM** | 1,416 (10.6%) | 322 (11.4%) | 87 (7.8%) | 140 (9.2%) | **0.003** |
| **GHTN** | 427 (3.2%) | 99 (3.5%) | 36 (3.2%) | 57 (3.7%) | 0.597 |
| **Pre-eclampsia** | 471 (3.5%) | 97 (3.4%) | 31 (2.8%) | 61 (4.0%) | 0.397 |
| **Eclampsia** | <5 (0.0%) | <5 (0.1%) | <5 (0.1%) | <5 (0.1%) | 0.068 |
| **Postpartum Haemorrhage** | 4,147 (30.9%) | 900 (31.8%) | 370 (33.1%) | 527 (34.6%) | **0.014** |
| Abbreviations: GDM = Gestational Diabetes Mellitus; PROM = Premature Rupture of Membranes; GHTN = Gestational Hypertension  * Pearson’s Chi-squared test; Fisher’s exact test | | | | | |

# Table S12 – Tests for effect modification between GDM and pregnancy outcomes

|  | **aOR (95% CI)*** | **p-value** | **Adjusted p-interaction Age** | **Adjusted p-interaction Ethnicity** | **Adjusted p-interaction IMD** |
| --- | --- | --- | --- | --- | --- |
| **Induction** | 1.53 (1.48, 1.57) | **<0.001** | **0.002** | 0.740 | 0.195 |
| **Mode of Delivery** |  |  |  |  |  |
| Elective Caesarean Section | 1.39 (1.34, 1.45) | **<0.001** | **0.002** | 0.740 | 0.751 |
| Emergency Caesarean Section | 1.18 (1.13, 1.22) | **<0.001** | **0.002** | 0.556 | 0.622 |
| Spontaneous Vaginal Birth | 0.77 (0.74, 0.79) | **<0.001** | **0.002** | 0.237 | 0.705 |
| Assisted Vaginal Birth | 0.90 (0.86, 0.94) | **<0.001** | **0.002** | 0.830 | 0.751 |
| **Preterm Delivery** | 1.31 (1.23, 1.39) | **<0.001** | 0.344 | 0.208 | 0.104 |
| **Stillbirth** | 0.51 (0.38, 0.68) | **<0.001** | 0.684 | 0.836 | 0.622 |
| **Polyhydramnios** | 2.41 (2.14, 2.72) | **<0.001** | 0.350 | 0.740 | 0.622 |
| **PROM** | 0.68 (0.65, 0.72) | **<0.001** | **0.002** | 0.691 | 0.705 |
| **GHTN** | 1.25 (1.14, 1.36) | **<0.001** | 0.791 | 0.691 | 0.745 |
| **Pre-eclampsia** | 1.19 (1.09, 1.30) | **<0.001** | 0.294 | 0.237 | 0.622 |
| **Eclampsia** | 1.14 (0.54, 2.14) | 0.707 | **0.037** | 0.691 | 0.622 |
| **Postpartum Haemorrhage** | 1.06 (1.03, 1.10) | **<0.001** | 0.525 | 0.052 | 0.273 |
| Abbreviations: GDM = Gestational Diabetes Mellitus; PROM = Premature Rupture of Membranes; GHTN = Gestational Hypertension, OR = Odds Ratio; aOR = Adjusted Odds Ratio; CI = Confidence Interval  * Adjusted for age at delivery, ethnicity, IMD 2019 quintile, and family history of diabetes  Interaction p-values derived from Wald tests of multiplicative interaction terms. | | | | | |

# Table S13 –- GEE-Estimated Associations Between First-time GDM, Ethnicity, and Obstetric Outcomes, Stratified by Body Mass Index

|  | **High BMI (≥30 kg/m²)** | | | **Low BMI (<30 kg/m²)** | | |
| --- | --- | --- | --- | --- | --- | --- |
|  | **aOR** | **95% CI** | **p** | **aOR** | **95% CI** | **p** |
| **Induction of Labour** | | | | | | |
| ***GDM Status (ref: no GDM)*** | | | | | | |
| First-time GDM | 1.47 | 1.34, 1.61 | **<0.001** | 1.55 | 1.49, 1.62 | **<0.001** |
| ***Ethnicity (ref: White)*** | | | | | | |
| Asian | 0.93 | 0.85, 1.01 | 0.104 | 1.14 | 1.11, 1.18 | **<0.001** |
| Black | 0.94 | 0.84, 1.04 | 0.211 | 1.07 | 1.02, 1.12 | **0.006** |
| Mixed | 0.86 | 0.72, 1.03 | 0.094 | 0.98 | 0.91, 1.05 | 0.516 |
| Other | 0.74 | 0.66, 0.83 | **<0.001** | 0.96 | 0.92, 1.00 | **0.045** |
| **Elective Caesarean Section** | | | | | | |
| ***GDM Status (ref: no GDM)*** | | | | | | |
| First-time GDM | 1.20 | 1.08, 1.34 | **0.001** | 1.34 | 1.26, 1.42 | **<0.001** |
| ***Ethnicity (ref: White)*** | | | | | | |
| Asian | 0.87 | 0.77, 0.97 | **0.011** | 0.74 | 0.71, 0.77 | **<0.001** |
| Black | 0.72 | 0.63, 0.83 | **<0.001** | 0.68 | 0.63, 0.73 | **<0.001** |
| Mixed | 0.85 | 0.67, 1.07 | 0.164 | 0.83 | 0.75, 0.92 | **<0.001** |
| Other | 1.07 | 0.93, 1.23 | 0.372 | 0.93 | 0.88, 0.99 | **0.020** |
| **Emergency Caesarean Section** | | | | | | |
| ***GDM Status (ref: no GDM)*** | | | | | | |
| First-time GDM | 1.33 | 1.21, 1.47 | **<0.001** | 1.14 | 1.09, 1.20 | **<0.001** |
| ***Ethnicity (ref: White)*** | | | | | | |
| Asian | 1.34 | 1.21, 1.47 | **<0.001** | 1.56 | 1.51, 1.61 | **<0.001** |
| Black | 1.29 | 1.15, 1.45 | **<0.001** | 1.42 | 1.35, 1.50 | **<0.001** |
| Mixed | 0.95 | 0.77, 1.16 | 0.608 | 1.22 | 1.12, 1.32 | **<0.001** |
| Other | 0.99 | 0.87, 1.13 | 0.911 | 1.12 | 1.07, 1.18 | **<0.001** |
| **Spontaneous Vaginal Birth** | | | | | | |
| ***GDM Status (ref: no GDM)*** | | | | | | |
| First-time GDM | 0.72 | 0.66, 0.79 | **<0.001** | 0.79 | 0.75, 0.82 | **<0.001** |
| ***Ethnicity (ref: White)*** | | | | | | |
| Asian | 0.76 | 0.70, 0.83 | **<0.001** | 0.79 | 0.77, 0.81 | **<0.001** |
| Black | 1.07 | 0.96, 1.19 | 0.246 | 1.20 | 1.15, 1.26 | **<0.001** |
| Mixed | 1.22 | 1.02, 1.46 | **0.033** | 1.09 | 1.02, 1.17 | **0.013** |
| Other | 0.98 | 0.88, 1.10 | 0.776 | 0.98 | 0.95, 1.03 | 0.458 |
| **Assisted Vaginal Birth** | | | | | | |
| ***GDM Status (ref: no GDM)*** | | | | | | |
| First-time GDM | 0.91 | 0.76, 1.08 | 0.275 | 1.00 | 0.94, 1.06 | 0.911 |
| ***Ethnicity (ref: White)*** | | | | | | |
| Asian | 1.43 | 1.24, 1.66 | **<0.001** | 1.13 | 1.09, 1.17 | **<0.001** |
| Black | 0.67 | 0.54, 0.84 | **<0.001** | 0.54 | 0.50, 0.59 | **<0.001** |
| Mixed | 0.74 | 0.51, 1.06 | 0.104 | 0.78 | 0.70, 0.86 | **<0.001** |
| Other | 0.91 | 0.74, 1.13 | 0.408 | 0.95 | 0.90, 1.01 | 0.097 |
| **Preterm Delivery** | | | | | | |
| ***GDM Status (ref: no GDM)*** | | | | | | |
| First-time GDM | 1.06 | 0.89, 1.26 | 0.533 | 1.33 | 1.23, 1.45 | **<0.001** |
| ***Ethnicity (ref: White)*** | | | | | | |
| Asian | 1.35 | 1.13, 1.60 | **<0.001** | 1.24 | 1.16, 1.32 | **<0.001** |
| Black | 1.24 | 1.00, 1.53 | **0.048** | 1.22 | 1.10, 1.35 | **<0.001** |
| Mixed | 0.71 | 0.46, 1.11 | 0.137 | 1.10 | 0.95, 1.27 | 0.209 |
| Other | 1.05 | 0.83, 1.33 | 0.707 | 0.88 | 0.80, 0.97 | **0.012** |
| **Stillbirth** | | | | | | |
| ***GDM Status (ref: no GDM)*** | | | | | | |
| First-time GDM | 0.55 | 0.25, 1.23 | 0.147 | 0.60 | 0.39, 0.90 | **0.015** |
| ***Ethnicity (ref: White)*** | | | | | | |
| White | — | — |  | — | — |  |
| Asian | 1.05 | 0.55, 2.00 | 0.877 | 1.57 | 1.24, 1.98 | **<0.001** |
| Black | 0.96 | 0.45, 2.03 | 0.910 | 1.63 | 1.16, 2.30 | **0.005** |
| Mixed | 0.75 | 0.17, 3.22 | 0.699 | 1.48 | 0.91, 2.43 | 0.117 |
| Other | 1.07 | 0.48, 2.39 | 0.872 | 1.00 | 0.70, 1.45 | 0.982 |
| **Polyhydramnios** | | | | | | |
| ***GDM Status (ref: no GDM)*** | | | | | | |
| First-time GDM | 2.06 | 1.52, 2.78 | **<0.001** | 2.60 | 2.19, 3.08 | **<0.001** |
| ***Ethnicity (ref: White)*** | | | | | | |
| Asian | 0.55 | 0.40, 0.76 | **<0.001** | 0.83 | 0.71, 0.98 | **0.026** |
| Black | 0.52 | 0.35, 0.78 | **0.002** | 1.35 | 1.07, 1.69 | **0.012** |
| Mixed | 0.65 | 0.33, 1.28 | 0.214 | 0.92 | 0.63, 1.32 | 0.638 |
| Other | 0.51 | 0.32, 0.81 | **0.004** | 1.07 | 0.86, 1.32 | 0.546 |
| **PROM** | | | | | | |
| ***GDM Status (ref: no GDM)*** | | | | | | |
| First-time GDM | 0.68 | 0.59, 0.79 | **<0.001** | 0.70 | 0.66, 0.75 | **<0.001** |
| ***Ethnicity (ref: White)*** | | | | | | |
| Asian | 1.25 | 1.10, 1.41 | **<0.001** | 1.34 | 1.29, 1.40 | **<0.001** |
| Black | 0.84 | 0.71, 0.98 | **0.028** | 0.79 | 0.73, 0.84 | **<0.001** |
| Mixed | 0.66 | 0.49, 0.89 | **0.007** | 0.88 | 0.79, 0.97 | **0.009** |
| Other | 0.87 | 0.74, 1.04 | 0.119 | 0.85 | 0.80, 0.90 | **<0.001** |
| **GHTN** | | | | | | |
| ***GDM Status (ref: no GDM)*** | | | | | | |
| First-time GDM | 1.08 | 0.87, 1.35 | 0.487 | 1.25 | 1.10, 1.43 | **<0.001** |
| ***Ethnicity (ref: White)*** | | | | | | |
| Asian | 0.65 | 0.53, 0.80 | **<0.001** | 0.97 | 0.89, 1.07 | 0.547 |
| Black | 0.73 | 0.57, 0.94 | **0.016** | 1.04 | 0.90, 1.21 | 0.565 |
| Mixed | 0.68 | 0.43, 1.06 | 0.086 | 0.72 | 0.56, 0.92 | **0.009** |
| Other | 0.55 | 0.41, 0.75 | **<0.001** | 0.77 | 0.67, 0.89 | **<0.001** |
| **Pre-eclampsia** | | | | | | |
| ***GDM Status (ref: no GDM)*** | | | | | | |
| First-time GDM | 1.06 | 0.85, 1.32 | 0.619 | 1.26 | 1.12, 1.42 | **<0.001** |
| ***Ethnicity (ref: White)*** | | | | | | |
| Asian | 1.04 | 0.84, 1.29 | 0.711 | 1.27 | 1.16, 1.38 | **<0.001** |
| Black | 1.09 | 0.84, 1.41 | 0.511 | 1.62 | 1.43, 1.85 | **<0.001** |
| Mixed | 0.90 | 0.57, 1.44 | 0.670 | 1.06 | 0.86, 1.31 | 0.598 |
| Other | 0.87 | 0.64, 1.16 | 0.343 | 0.92 | 0.81, 1.06 | 0.247 |
| **Eclampsia** | | | | | | |
| ***GDM Status (ref: no GDM)*** | | | | | | |
| First-time GDM | 7.58 | 1.39, 41.3 | **0.019** | 0.92 | 0.35, 2.46 | 0.872 |
| ***Ethnicity (ref: White)*** | | | | | | |
| Asian | 1.72 | 0.15, 19.1 | 0.660 | 1.45 | 0.79, 2.67 | 0.228 |
| Black | 2.28 | 0.15, 33.8 | 0.549 | 0.55 | 0.13, 2.32 | 0.415 |
| Mixed | 9.27 | 0.42, 205 | 0.159 | 2.89 | 1.09, 7.66 | **0.033** |
| Other | 2.13 | 0.14, 32.9 | 0.590 | 1.23 | 0.52, 2.95 | 0.635 |
| **Postpartum Haemorrhage** | | | | | | |
| ***GDM Status (ref: no GDM)*** | | | | | | |
| First-time GDM | 1.04 | 0.95, 1.14 | 0.422 | 1.04 | 1.00, 1.09 | 0.066 |
| ***Ethnicity (ref: White)*** | | | | | | |
| Asian | 1.16 | 1.06, 1.27 | **0.001** | 1.19 | 1.15, 1.23 | **<0.001** |
| Black | 0.99 | 0.89, 1.10 | 0.835 | 0.93 | 0.88, 0.98 | **0.006** |
| Mixed | 0.89 | 0.74, 1.08 | 0.236 | 0.99 | 0.92, 1.06 | 0.732 |
| Other | 0.97 | 0.86, 1.09 | 0.609 | 1.04 | 0.99, 1.08 | 0.097 |

Abbreviations: aOR = Adjusted Odds Ratio; CI = Confidence Interval; GDM = Gestational Diabetes Mellitus; GEE = Generalised Estimating Equations; BMI = Body Mass Index; PROM = Premature Rupture of Membranes; GHTN = Gestational Hypertension.

Models were fitted separately within each BMI stratum (high, 30 kg/m² and above; low, below 30 kg/m²) using GEE with an exchangeable correlation structure to account for repeated births per woman. Each model was adjusted for maternal age at delivery, ethnicity, IMD quintile, and family history of diabetes.

Reference categories: no GDM for GDM type and White for ethnicity

# Table S14 –- GEE-Estimated Associations Between First-time GDM, Ethnicity, and Obstetric Outcomes, Stratified by Deprivation Index (IMD)

|  | **High deprivation (IMD 1-2)** | | | **Low deprivation (IMD 3-5)** | | |
| --- | --- | --- | --- | --- | --- | --- |
|  | **aOR** | **95% CI** | **p** | **aOR** | **95% CI** | **p** |
| **Induction of Labour** | | | | | | |
| ***GDM Status (ref: no GDM)*** | | | | | | |
| First-time GDM | 1.62 | 1.54, 1.69 | **<0.001** | 1.53 | 1.46, 1.60 | **<0.001** |
| ***Ethnicity (ref: White)*** | | | | | | |
| Asian | 1.10 | 1.06, 1.14 | **<0.001** | 1.15 | 1.12, 1.19 | **<0.001** |
| Black | 1.00 | 0.96, 1.05 | 0.843 | 1.11 | 1.04, 1.18 | **0.001** |
| Mixed | 0.96 | 0.89, 1.04 | 0.306 | 0.97 | 0.89, 1.05 | 0.457 |
| Other | 0.87 | 0.83, 0.92 | **<0.001** | 0.99 | 0.95, 1.04 | 0.705 |
| **Elective Caesarean Section** | | | | | | |
| ***GDM Status (ref: no GDM)*** | | | | | | |
| First-time GDM | 1.37 | 1.29, 1.45 | **<0.001** | 1.31 | 1.23, 1.39 | **<0.001** |
| ***Ethnicity (ref: White)*** | | | | | | |
| Asian | 0.77 | 0.73, 0.81 | **<0.001** | 0.75 | 0.72, 0.79 | **<0.001** |
| Black | 0.76 | 0.71, 0.81 | **<0.001** | 0.72 | 0.65, 0.79 | **<0.001** |
| Mixed | 0.83 | 0.74, 0.93 | **0.001** | 0.87 | 0.78, 0.98 | **0.016** |
| Other | 1.04 | 0.97, 1.11 | 0.253 | 0.92 | 0.86, 0.98 | **0.010** |
| **Emergency Caesarean Section** | | | | | | |
| ***GDM Status (ref: no GDM)*** | | | | | | |
| First-time GDM | 1.18 | 1.12, 1.24 | **<0.001** | 1.24 | 1.17, 1.31 | **<0.001** |
| ***Ethnicity (ref: White)*** | | | | | | |
| Asian | 1.55 | 1.48, 1.61 | **<0.001** | 1.64 | 1.58, 1.71 | **<0.001** |
| Black | 1.38 | 1.31, 1.46 | **<0.001** | 1.59 | 1.48, 1.71 | **<0.001** |
| Mixed | 1.18 | 1.08, 1.29 | **<0.001** | 1.27 | 1.16, 1.40 | **<0.001** |
| Other | 1.08 | 1.02, 1.14 | **0.010** | 1.16 | 1.10, 1.23 | **<0.001** |
| **Spontaneous Vaginal Birth** | | | | | | |
| ***GDM Status (ref: no GDM)*** | | | | | | |
| First-time GDM | 0.78 | 0.75, 0.82 | **<0.001** | 0.76 | 0.73, 0.80 | **<0.001** |
| ***Ethnicity (ref: White)*** | | | | | | |
| Asian | 0.74 | 0.72, 0.77 | **<0.001** | 0.78 | 0.76, 0.81 | **<0.001** |
| Black | 1.14 | 1.09, 1.20 | **<0.001** | 1.09 | 1.02, 1.17 | **0.008** |
| Mixed | 1.11 | 1.03, 1.20 | **0.009** | 1.02 | 0.93, 1.10 | 0.714 |
| Other | 0.97 | 0.93, 1.02 | 0.288 | 0.97 | 0.92, 1.01 | 0.158 |
| **Assisted Vaginal Birth** | | | | | | |
| ***GDM Status (ref: no GDM)*** | | | | | | |
| First-time GDM | 0.98 | 0.91, 1.05 | 0.501 | 0.95 | 0.89, 1.02 | 0.177 |
| ***Ethnicity (ref: White)*** | | | | | | |
| Asian | 1.21 | 1.15, 1.27 | **<0.001** | 1.09 | 1.05, 1.14 | **<0.001** |
| Black | 0.52 | 0.48, 0.56 | **<0.001** | 0.53 | 0.48, 0.59 | **<0.001** |
| Mixed | 0.75 | 0.67, 0.84 | **<0.001** | 0.80 | 0.71, 0.90 | **<0.001** |
| Other | 0.90 | 0.85, 0.97 | **0.003** | 0.96 | 0.90, 1.02 | 0.230 |
| **Preterm Delivery** | | | | | | |
| ***GDM Status (ref: no GDM)*** | | | | | | |
| First-time GDM | 1.20 | 1.10, 1.32 | **<0.001** | 1.44 | 1.32, 1.58 | **<0.001** |
| ***Ethnicity (ref: White)*** | | | | | | |
| Asian | 1.27 | 1.18, 1.37 | **<0.001** | 1.27 | 1.19, 1.37 | **<0.001** |
| Black | 1.18 | 1.07, 1.30 | **<0.001** | 1.31 | 1.15, 1.49 | **<0.001** |
| Mixed | 1.11 | 0.94, 1.30 | 0.218 | 1.03 | 0.86, 1.24 | 0.732 |
| Other | 0.87 | 0.79, 0.97 | **0.014** | 1.01 | 0.91, 1.12 | 0.839 |
| **Stillbirth** | | | | | | |
| ***GDM Status (ref: no GDM)*** | | | | | | |
| First-time GDM | 0.59 | 0.39, 0.87 | **0.009** | 0.41 | 0.23, 0.71 | **0.002** |
| ***Ethnicity (ref: White)*** | | | | | | |
| Asian | 1.62 | 1.24, 2.11 | **<0.001** | 1.41 | 1.09, 1.82 | **0.009** |
| Black | 1.83 | 1.35, 2.49 | **<0.001** | 1.31 | 0.80, 2.13 | 0.283 |
| Mixed | 1.59 | 0.97, 2.60 | 0.066 | 0.92 | 0.45, 1.88 | 0.812 |
| Other | 1.02 | 0.70, 1.51 | 0.900 | 1.24 | 0.86, 1.78 | 0.247 |
| **Polyhydramnios** | | | | | | |
| ***GDM Status (ref: no GDM)*** | | | | | | |
| First-time GDM | 2.43 | 2.05, 2.89 | **<0.001** | 2.51 | 2.08, 3.01 | **<0.001** |
| ***Ethnicity (ref: White)*** | | | | | | |
| Asian | 0.75 | 0.63, 0.89 | **0.001** | 0.87 | 0.73, 1.03 | 0.095 |
| Black | 0.93 | 0.75, 1.15 | 0.480 | 1.41 | 1.07, 1.84 | **0.014** |
| Mixed | 1.02 | 0.73, 1.43 | 0.887 | 0.62 | 0.38, 1.02 | 0.059 |
| Other | 0.84 | 0.67, 1.04 | 0.114 | 1.04 | 0.83, 1.30 | 0.764 |
| **PROM** | | | | | | |
| ***GDM Status (ref: no GDM)*** | | | | | | |
| First-time GDM | 0.70 | 0.65, 0.76 | **<0.001** | 0.72 | 0.67, 0.78 | **<0.001** |
| ***Ethnicity (ref: White)*** | | | | | | |
| Asian | 1.32 | 1.26, 1.38 | **<0.001** | 1.35 | 1.29, 1.41 | **<0.001** |
| Black | 0.80 | 0.74, 0.85 | **<0.001** | 0.76 | 0.69, 0.84 | **<0.001** |
| Mixed | 0.81 | 0.72, 0.90 | **<0.001** | 0.94 | 0.84, 1.05 | 0.290 |
| Other | 0.81 | 0.75, 0.86 | **<0.001** | 0.87 | 0.81, 0.93 | **<0.001** |
| **GHTN** | | | | | | |
| ***GDM Status (ref: no GDM)*** | | | | | | |
| First-time GDM | 1.24 | 1.08, 1.41 | **0.002** | 1.22 | 1.07, 1.40 | **0.004** |
| ***Ethnicity (ref: White)*** | | | | | | |
| Asian | 0.96 | 0.86, 1.06 | 0.413 | 0.91 | 0.83, 1.01 | 0.075 |
| Black | 0.95 | 0.83, 1.09 | 0.433 | 1.24 | 1.04, 1.47 | **0.017** |
| Mixed | 0.77 | 0.60, 0.99 | 0.044 | 0.72 | 0.54, 0.96 | 0.028 |
| Other | 0.70 | 0.60, 0.82 | **<0.001** | 0.76 | 0.65, 0.88 | **<0.001** |
| **Pre-eclampsia** | | | | | | |
| ***GDM Status (ref: no GDM)*** | | | | | | |
| First-time GDM | 1.22 | 1.09, 1.38 | **<0.001** | 1.27 | 1.12, 1.45 | **<0.001** |
| ***Ethnicity (ref: White)*** | | | | | | |
| Asian | 1.29 | 1.17, 1.43 | **<0.001** | 1.24 | 1.12, 1.37 | **<0.001** |
| Black | 1.39 | 1.23, 1.58 | **<0.001** | 1.73 | 1.47, 2.05 | **<0.001** |
| Mixed | 1.01 | 0.81, 1.26 | 0.946 | 1.03 | 0.80, 1.33 | 0.834 |
| Other | 0.86 | 0.74, 0.99 | **0.038** | 1.01 | 0.87, 1.17 | 0.901 |
| **Eclampsia** | | | | | | |
| ***GDM Status (ref: no GDM)*** | | | | | | |
| First-time GDM | 2.41 | 1.05, 5.53 | **0.039** | 0.60 | 0.18, 2.00 | 0.409 |
| ***Ethnicity (ref: White)*** | | | | | | |
| Asian | 0.96 | 0.45, 2.04 | 0.907 | 1.31 | 0.66, 2.61 | 0.437 |
| Black | 1.13 | 0.43, 3.01 | 0.801 | 1.42 | 0.42, 4.75 | 0.570 |
| Mixed | 1.20 | 0.27, 5.40 | 0.808 | 3.24 | 1.10, 9.57 | **0.033** |
| Other | 1.06 | 0.39, 2.84 | 0.914 | 1.56 | 0.64, 3.78 | 0.327 |
| **Postpartum Haemorrhage** | | | | | | |
| ***GDM Status (ref: no GDM)*** | | | | | | |
| First-time GDM | 1.07 | 1.02, 1.12 | **0.008** | 1.10 | 1.05, 1.16 | **<0.001** |
| ***Ethnicity (ref: White)*** | | | | | | |
| Asian | 1.19 | 1.14, 1.23 | **<0.001** | 1.20 | 1.16, 1.24 | **<0.001** |
| Black | 0.94 | 0.89, 0.99 | **0.012** | 0.98 | 0.91, 1.05 | 0.555 |
| Mixed | 0.98 | 0.91, 1.07 | 0.680 | 0.98 | 0.90, 1.07 | 0.695 |
| Other | 1.00 | 0.95, 1.05 | 0.997 | 1.05 | 1.00, 1.10 | **0.044** |

Abbreviations: aOR = Adjusted Odds Ratio; CI = Confidence Interval; GDM = Gestational Diabetes Mellitus; GEE = Generalised Estimating Equations; IMD = Index of Multiple Deprivation; PROM = Premature Rupture of Membranes; GHTN = Gestational Hypertension.

High deprivation comprises IMD quintiles 1 to 2 and low deprivation quintiles 3 to 5. Models were fitted separately within each deprivation stratum using GEE with an exchangeable correlation structure. Each model was adjusted for maternal age at delivery, ethnicity, and family history of diabetes (IMD was omitted as the stratifying variable).

Reference categories: no GDM for GDM type and White for ethnicity.

# Table S15 – Obstetric Outcomes by first-time GDM vs recurrent-GDM pregnancy

|  | **First time GDM N=16,725** | **Recurrent GDM N=2,167** | **p-value** |
| --- | --- | --- | --- |
| **Induction** | 8,686 (51.9%) | 938 (43.3%) | **<0.001** |
| **Mode of Delivery** |  |  |  |
| Elective Caesarean Section | 3,305 (19.8%) | 520 (24.0%) | **<0.001** |
| Emergency Caesarean Section | 4,633 (27.7%) | 399 (18.4%) | **<0.001** |
| Spontaneous Vaginal Birth | 6,597 (39.4%) | 1,142 (52.7%) | **<0.001** |
| Assisted Vaginal Birth | 2,190 (13.1%) | 106 (4.9%) | **<0.001** |
| **Preterm Delivery** | 1,228 (7.3%) | 143 (6.6%) | 0.209 |
| **Stillbirth** | 43 (0.3%) | 5 (0.2%) | 0.819 |
| **Polyhydramnios** | 356 (2.1%) | 37 (1.7%) | 0.196 |
| **PROM** | 1,827 (10.9%) | 138 (6.4%) | **<0.001** |
| **GHTN** | 547 (3.3%) | 72 (3.3%) | 0.898 |
| **Pre-eclampsia** | 619 (3.7%) | 41 (1.9%) | **<0.001** |
| **Eclampsia** | 10 (0.1%) | 0 (0.0%) | 0.617 |
| **Postpartum Haemorrhage** | 5,370 (32.1%) | 574 (26.5%) | **<0.001** |
| Abbreviations: GDM = Gestational Diabetes Mellitus; PROM = Premature Rupture of Membranes; GHTN = Gestational Hypertension | | | |

# Figure S1 -– Cohort derivation flow diagram

# Figure S2 – Prevalence (%) of outcomes by Age group and GDM status

# Figure S3 – Prevalence (%) of outcomes by Ethnicity and GDM status

# Figure S4 – Prevalence (%) of outcomes by IMD quintile and GDM status
